# Multimorbidity and healthcare resource utilization in Switzerland: a multicentre cohort study

**DOI:** 10.1186/s12913-019-4575-2

**Published:** 2019-10-17

**Authors:** Carole E. Aubert, Niklaus Fankhauser, Pedro Marques-Vidal, Jérôme Stirnemann, Drahomir Aujesky, Andreas Limacher, Jacques Donzé

**Affiliations:** 1 Department of General Internal Medicine, Inselspital, Bern University Hospital, University of Bern, Freiburgstrasse, CH-3010 Bern, Switzerland; 2 Institute of Primary Health Care (BIHAM), University of Bern, Bern, Switzerland; 3 CTU, University of Bern, Bern, Switzerland; 4 Department of Internal Medicine, Lausanne University Hospital, Lausanne, Switzerland; 5 Department of Internal Medicine, Geneva University Hospitals, Geneva, Switzerland; 6 Division of General Medicine, BWH Hospitalist Service, Brigham and Women’s Hospital, Boston, MA USA; 7 Harvard Medical School, Boston, MA USA; 8 Department of Internal Medicine, Hôpital neuchâtelois, Neuchâtel, Switzerland

**Keywords:** Multimorbidity, Comorbidity, Chronic diseases, Combinations, Readmission, Potentially avoidable readmission, Length of stay, Healthcare utilization

## Abstract

**Background:**

Multimorbidity is associated with higher healthcare resource utilization, but we lack data on the association of specific combinations of comorbidities with healthcare resource utilization. We aimed to identify the combinations of comorbidities associated with high healthcare resource utilization among multimorbid medical inpatients.

**Methods:**

We performed a multicentre retrospective cohort study including 33,871 multimorbid (≥2 chronic diseases) medical inpatients discharged from three Swiss hospitals in 2010–2011. Healthcare resource utilization was measured as 30-day potentially avoidable readmission (PAR), prolonged length of stay (LOS) and difference in median LOS. We identified the combinations of chronic comorbidities associated with the highest healthcare resource utilization and quantified this association using regression techniques.

**Results:**

Three-fourths of the combinations with the strongest association with PAR included chronic kidney disease. Acute and unspecified renal failure combined with solid malignancy was most strongly associated with PAR (OR 2.64, 95%CI 1.79;3.90). Miscellaneous mental health disorders combined with mood disorders was the most strongly associated with LOS (difference in median LOS: 17 days) and prolonged LOS (OR 10.77, 95%CI 8.38;13.84). The number of chronic diseases was strongly associated with prolonged LOS (OR 9.07, 95%CI 8.04;10.24 for ≥10 chronic diseases), and to a lesser extent with PAR (OR 2.16, 95%CI 1.75;2.65 for ≥10 chronic diseases).

**Conclusions:**

Multimorbidity appears to have a higher impact on LOS than on PAR. Combinations of comorbidities most strongly associated with healthcare utilization included kidney disorders for PAR, and mental health disorders for LOS.

## Background

With the progress of medical science, survival has increased for patients with several chronic diseases, leading to a higher prevalence of multimorbidity, most often defined as the presence of at least two chronic diseases [1–3]. Given its association with poor quality of life, functional decline and healthcare resource utilization, multimorbidity represents a significant burden for the healthcare systems and the patients [4–6].

Potentially avoidable readmission (PAR) and length of stay (LOS) are two different and relatively simple measures of healthcare resource utilization among inpatients [7]. Given that both the rate of PAR and the LOS can be effectively reduced by various preventive but often complex and costly interventions, it is essential to identify patients at higher risk for PAR or prolonged LOS, in order to improve allocation of these interventions [8–10]. While previous studies conducted mainly in the United States, but also Australia, Ireland and Spain, described an association between multimorbidity and readmission or prolonged LOS [11–18], data in Switzerland are scarce, with only two studies showing a prolonged LOS in patients with multimorbidity [1–3]. Nevertheless, we lack descriptive and more detailed quantitative data on the association between particular combinations of chronic comorbidities and PAR or prolonged LOS. Furthermore, more complex effects of chronic comorbidities on the odds for PAR or prolonged LOS, such as interactions that may lead to more than multiplicative effects on this odds, have not yet been studied.

Our primary aim was to identify combinations of chronic comorbidities associated with higher healthcare resource utilization, measured as PAR and prolonged LOS, to quantify this association, and to identify potential interactions between those comorbidities on the odds for PAR or prolonged LOS. Our hypothesis was that particular combinations of comorbidities are associated with a higher odds for PAR or prolonged LOS, and that some comorbidities found in those combinations may have a more or a less than multiplicative effect on this odds. Our secondary aim was to quantify the association between the number of chronic diseases and healthcare resource utilization. Our hypothesis was that a higher number of chronic diseases is associated with higher odds for PAR and prolonged LOS.

## Methods

### Study design, setting, and participants

We used a retrospective cohort including all consecutive patients with multimorbidity – defined by its most usual definition, i.e. the presence of at least two chronic diseases [3, 19] – who were discharged home or to a nursing home from the medical departments of three large university teaching hospitals in Switzerland (Bern University Hospital [Inselspital], Lausanne University Hospital [CHUV], and Geneva University Hospitals [HUG]) between 2010 and 2011. These three hospitals are comparable in terms of size, types of patients cared for and treatment approaches. This is an ancillary study using only the Swiss sample from a larger multinational cohort initially developed to study readmission in four countries (Canada, USA, Switzerland, Israel) [20]. The unit of analysis was hospital discharge. All data were extracted from hospital electronic medical records. We did not assess death events following discharge. As the data were fully anonymised, an ethical approval was not required according to Swissethics. Reporting is in accordance with the STrengthening the Reporting of OBservational studies in Epidemiology (STROBE) statement [21].

### Classification of diseases

We used the Chronic Condition Indicator, developed by the Healthcare Cost and Utilization Project, a Federal-State-Industry partnership sponsored by the Agency for Healthcare Research and Quality, to differentiate between chronic and not chronic diseases [22]. According to this standardised tool, International Classification of Diseases (ICD) diagnosis codes are classified as chronic if they meet following criteria: a health condition lasting at least 12 months *and* causing limitations on self-care, independent living and social interactions, *and/or* resulting in the need for ongoing intervention with medical products, services and special equipment. To focus on conditions that define multimorbidity, we included only chronic diseases in our analysis, and thus excluded ICD codes for acute diseases, risk factors, symptoms, screening strategies and complications of diseases, as previously done [23]. We then collapsed all chronic diseases into a clinically meaningful number of categories using the Clinical Classification Software, also developed by the Healthcare Cost and Utilization Project [24], defining the different chronic comorbidities to be analysed. We finally grouped together some of those comorbidities that were expected to be found in combination (details in Additional file 1).

### Study outcomes

Our primary outcome was potentially avoidable readmission (PAR) to any department of the same hospital within 30 days following hospital discharge. To identify PAR, we used the SQLape algorithm, which is based on administrative data and ICD codes and was initially developed in Switzerland to benchmark hospitals [20, 25]. Shortly, this algorithm classifies a readmission as unavoidable if it was predictable, for example for planed chemotherapy or radiotherapy, or if it involves a new body system not affected during index admission. Readmissions for transplantation, severe trauma, severe chronic disease or rehabilitation treatment are also considered unavoidable. On the other hand, readmission for complications is deemed avoidable. Our secondary outcome was a prolonged LOS, assessed both as a continuous and binary variable. For the binary assessment, we defined a prolonged LOS as a LOS longer or equal to the upper quartile (75%).

### Statistical analyses

We presented patients’ baseline characteristics as median with interquartile range (IQR) or proportions, as appropriate.

For the primary aim, we used a mixed-effects logistic regression adjusted for age and sex, with a random intercept for hospital to identify the 20 combinations of comorbidities with the highest odds for PAR and for a prolonged LOS. For clinical relevance, we only considered combinations with a prevalence of ≥0.5%. We presented the results as odds ratio (OR) with 95% confidence interval (CI). We compared the patients with, to the patients without the combinations of comorbidities. For each of these 20 combinations, we then analysed interactions to identify the comorbidities that had a more or a less than multiplicative effect on the odds for PAR or prolonged LOS. A more than multiplicative effect means that the OR of the combination is higher than the ORs of the single comorbidities multiplied together. A less than multiplicative effect means the opposite. We used quantile regression controlled for age, sex and hospital to assess the difference in median LOS between patients with and without a combination of comorbidities.

For the secondary aim, we used a mixed-effects logistic univariable regression with a random intercept for centre to quantify the association between the number of chronic diseases and PAR or prolonged LOS, respectively. We used a forest plot to present the results.

We performed all analyses with STATA 15.1 (StataCorp LP, College Station, TX, USA) or R version 3.4.4 (R Project for Statistical Computing).

## Results

**Fig. 1:**
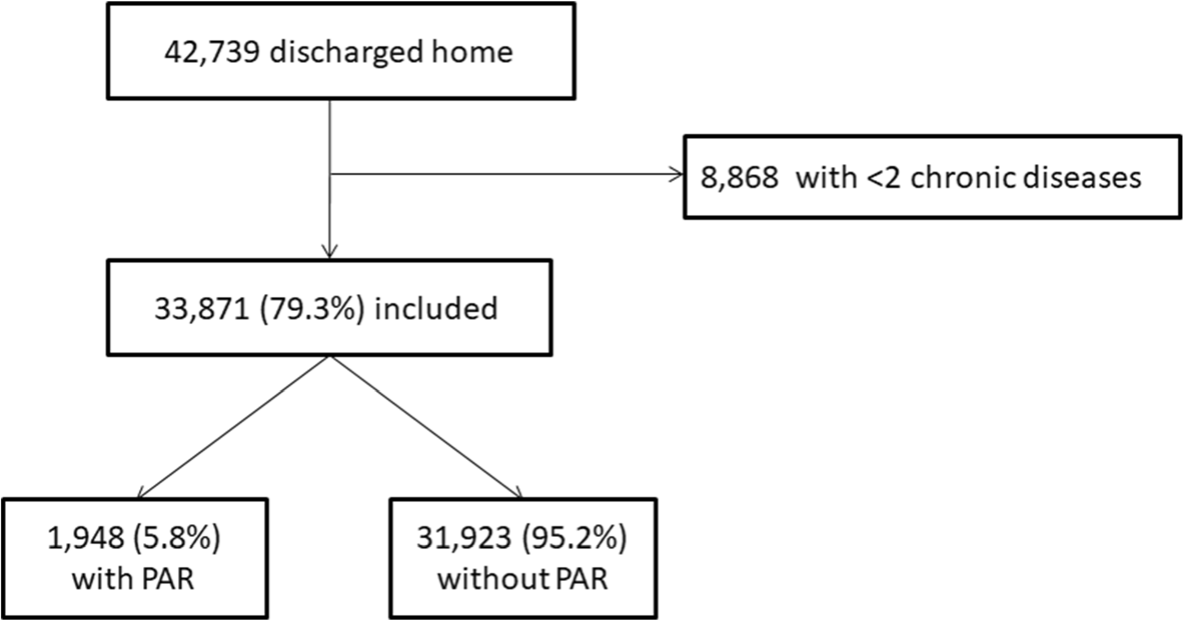
Study flow-chart. Abbreviations: PAR, potentially avoidable readmission

Among the 42,739 patients available in the initial cohort, 33,871 (79.3%) were multimorbid and included in the study (Fig. 1). Among them, 1948 (5.8%) had a PAR within 30 days after discharge, and 9634 (28.4%) had a prolonged LOS. Median age was 68 years (IQR 56–78), median number of chronic diseases was 4 (IQR 3–6), and median LOS was 8 days (IQR 4–14; Table 1). Median LOS was 5 days (IQR 2–8) in patients without prolonged LOS and 18 days (IQR 15–25) in those with prolonged LOS. The most frequent comorbidities were chronic heart disease, chronic kidney disease (CKD), solid malignancy and substance-related disorders, each occurring in more than 10% of the patients.

**Table 1:** Baseline characteristics

Characteristics	Multimorbid population (*n* = 33,871)
Age, years, median (IQR)	68 (56–78)
Men, n (%)	19,170 (56.6)
Length of stay, days, median (IQR)	8 (4–14)
Potentially avoidable readmission, n (%)	1948 (5.8)
Number of admissions in the past year, median (IQR)	0 (0–2)
*Description of multimorbidity*	
Number of acute and chronic diseases, median (IQR)	7 (5–10)
Number of chronic diseases, median (IQR)	4 (3–6)
Deyo-Charlson Comorbidity Index, median (IQR)^a^	2 (1–3)
Elixhauser-Van Walraven Comorbidity Index, median (IQR)^b^	7 (2–12)
*Most frequent comorbidities (prevalence > 10%)*	
Chronic heart disease, n (%)	15,717 (46.4)
Chronic kidney disease, n (%)	5174 (15.3)
Solid malignancy, n (%)	4964 (14,7)
Substance-related disorders, n (%)	4153 (12.3)

*Abbreviations*: *IQR* interquartile range, *n* number

^a^A prolonged length of stay was defined as a stay longer than or equal to upper quartile (75%)

^a^Deyo-Charlson Comorbidity Index ranges from 0 to + 33

^b^Elixhauser-Van Walraven Comorbidity Index ranges from − 19 to + 86

### Combinations of comorbidities and 30-day potentially avoidable readmission

Among the 20 combinations of comorbidities with the highest OR for PAR, 10 included CKD and 7 included solid malignancy (Table 2). Acute and unspecified renal failure combined with solid malignancy showed the highest OR for PAR (OR 2.69, 95%CI 1.81;3.99), followed by pulmonary heart disease combined with solid malignancy (OR 2.48, 95% CI 1.70;3.63, Table 2). The 18 following combinations increased the odds for PAR by 92 to 140%. CKD in combination with other diseases of kidney and ureters, and CKD in combination with solid malignancy had a less than multiplicative effect on the odds for PAR. Paralysis in combination with other nervous system disorders had a more than multiplicative effect on the odds for PAR, while the comorbidities of the other 17 combinations showed no significant interaction (Table 2).

**Table 2:** Combinations of comorbidities and 30-day potentially avoidable readmission

Combinations of comorbidities ^a^		OR (95% CI)	Interaction ^b^
Acute and unspecified renal failure	Solid malignancy	2.69 (1.81;3.99)	0
Pulmonary heart disease	Solid malignancy	2.48 (1.70;3.63)	0
Chronic kidney disease	Liver disease	2.40 (1.60;3.60)	–
Chronic kidney disease	Solid malignancy	2.39 (1.79;3.20)	0
Solid malignancy	Chronic obstructive pulmonary disease and bronchiectasis	2.36 (1.75;3.17)	0
Chronic kidney disease	Chronic obstructive pulmonary disease and bronchiectasis	2.29 (1.76;2.97)	0
Chronic kidney disease	Other nutritional, endocrine or metabolic disorders	2.23 (1.41;3.54)	0
Chronic kidney disease	Pulmonary heart disease	2.15 (1.64;2.83)	0
Esophageal disorders	Solid malignancy	2.15 (1.39; 3.33)	0
Liver disease	Solid malignancy	2.13 (1.41; 3.22)	0
Chronic kidney disease	Other and ill-defined heart disease	2.13 (1.45;3.13)	0
Chronic kidney disease	Other diseases of kidney and ureters	2.11 (1.41;3.16)	–
Chronic kidney disease	Thyroid disorders	2.09 (1.53;2.85)	0
Pulmonary heart disease	Chronic obstructive pulmonary disease and bronchiectasis	2.06 (1.51;2.80)	0
Chronic kidney disease	Chronic heart disease	2.02 (1.77;2.30)	0
Chronic heart disease	Systemic lupus erythematosus and connective tissue disorders	2.01 (1.26;3.21)	0
Paralysis	Other nervous system disorders	1.98 (1.39;2.82)	+
Chronic kidney disease	Nephritis, nephrosis, renal sclerosis	1.95 (1.55;2.47)	0
Epilepsy; convulsions	Solid malignancy	1.92 (1.25;2.95)	0
Chronic heart disease	Other lower respiratory disease	1.92 (1.18;3.13)	0

*Abbreviations*: *CI* confidence interval, *OR* odds ratio, *PAR* potentially avoidable readmission

^a^20 combinations with the highest OR for PAR

^b^ The sign "+"represents a significant more than multiplicative effect of the comorbidities found in the combination on the odds for PAR. A more than multiplicative effect means that the OR of the combination is larger than the ORs of the single comorbidities multiplied together. The sign "-"represents a significant less than multiplicative effect of the comorbidities found in the combination on the odds for PAR. A less than multiplicative effect means that the OR of the combination is smaller than the ORs of the single comorbidities multiplied together. The sign "0"means that there is no significant interaction

### Combinations of comorbidities and prolonged length of stay

Two combinations of comorbidities showed a particular high OR for a prolonged LOS and a particular high difference in median LOS (Table 3): 1) miscellaneous mental health disorders combined with mood disorders (OR for prolonged LOS: 11.28, 95%CI 8.71;14.61; difference in median LOS: 17.4 days, 95%CI 16.5;18.3); 2) diseases of white blood cells combined with hematological malignancy (OR for prolonged LOS: 10.95, 95%CI 9.06;13.24; difference in median LOS: 15.7 days, 95%CI 15.1;16.4).

**Table 3:** Combinations of comorbidities and prolonged length of stay

Combinations of comorbidities ^a^		OR (95% CI) ^b^	Interaction ^c^	Difference in median LOS (95% CI) ^d^
Miscellaneous mental health disorders	Mood disorders	11.28 (8.71;14.61)	+	17.3 (16.5;18.3)
Diseases of white blood cells	Hematological malignancy	10.95 (9.06;13.24)	+	15.7 (15.1;16.8)
Chronic heart disease	Chronic ulcer of skin	4.83 (3.77;6.17)	+	7.8 (6.8;8.7)
Anxiety disorders	Mood disorders	4.10 (3.06;5.49)	0	7.6 (6.4;8.8)
Chronic heart disease	Diseases of white blood cells	3.92 (3.01;5.11)	0	8.0 (7.0;9.1)
Paralysis	Solid malignancy	3.37 (2.54;4.47)	0	6.6 (5.5;7.8)
Chronic heart disease	Liver disease	3.36 (2.76;4.08)	+	6.2 (5.5;7.0)
Chronic kidney disease	Chronic ulcer of skin	3.22 (2.40;4.31)	–	5.4 (4.2;6.5)
Chronic heart disease	Hematological malignancy	3.21 (2.62;3.92)	0	5.2 (4.3;6.0)
Chronic kidney disease	Hematological malignancy	3.16 (2.42;4.14)	0	4.3 (3.2;5.4)
Arthropathy and arthritis	Mood disorders	3.10 (2.42;9.96)	–	5.7 (4.8;6.7)
Paralysis	Other nervous system disorders	3.02 (2.40;3.78)	0	5.1 (4.2;6.0)
Miscellaneous mental health disorders	Chronic heart disease	3.01 (2.30;3.93)	0	4.8 (3.8;5.9)
Dementia	Mood disorders	2.95 (2.27;3;82)	–	5.6 (4.6;6.6)
Paralysis	Epilepsy; convulsions	2.92 (2.28;3.75)	0	5.0 (4.0;6.0)
Other nervous system disorders	Mood disorders	2.89 (2.28;3.66)	0	5.9 (5.0;6.9)
Chronic kidney disease	Liver disease	2.89 (2.19;3.80)	0	4.9 (3.8;6.1)
Dementia	Substance-related disorders	2.83 (2.13;3.75)	0	5.1 (3.9;6.2)
Other nervous system disorders	Cerebrovascular disease	2.79 (2.21;3.54)	0	4.6 (3.7;5.6)
Epilepsy; convulsions	Cerebrovascular disease	2.79 (2.09;3.73)	+	5.3 (4.2;6.5)

*Abbreviations*: *CI* confidence interval, *LOS* length of stay, *OR* odds ratio

^a^20 combinations with the highest OR for PAR

^b^A prolonged length of stay was defined as a stay longer than or equal to the upper quartile (75%)

^c^The signs "+" and "-"represent a significant more than multiplicative effect and less than multiplicative effect, respectively, of the comorbidities found in the combination on the odds for a prolonged LOS. A more than multiplicative effect means that the OR of the combination is higher than the ORs of the single comorbidities multiplied together. The sign "0"means that there is no significant interaction

^d^*p*-value < 0.001 for all. A confidence interval not overlapping the zero line means that the difference in median is significant

The 18 following combinations increased the odds for a prolonged LOS by 179 to 383% and showed a difference in median LOS of 4 to 8 days. These combinations included mostly mental health conditions, neurological diseases and renal diseases. The comorbidities of five combinations had a more than multiplicative effect on the odds for a prolonged LOS, while those of three combinations had a less than multiplicative effect on this odds (Table 3).

### Number of chronic diseases and healthcare resource utilization

**Fig. 2:**
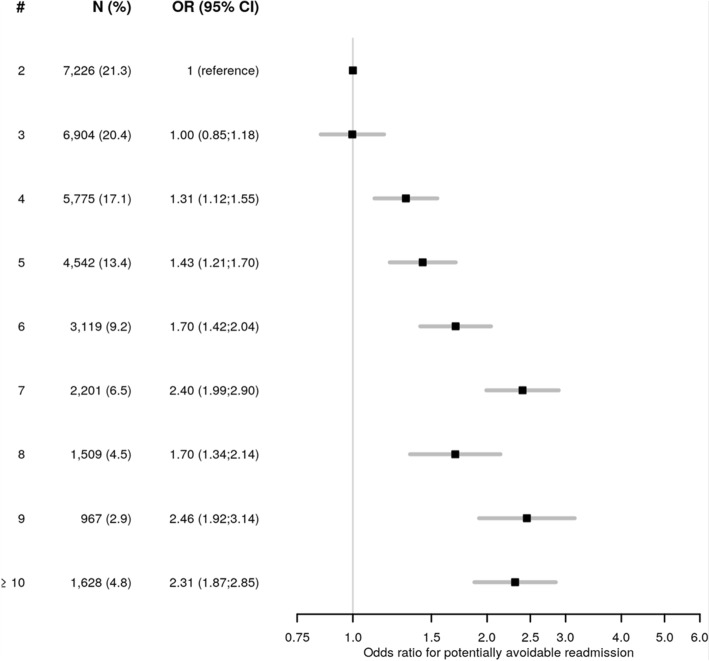
Association between the number of chronic diseases and 30-day potentially avoidable readmission. Legend: Odds ratio (box) with 95% confidence interval (lines) for 30-day potentially avoidable readmission. The reference category is the presence of 2 chronic diseases, as we included only patients with multimorbidity. Abbreviations: #, number of chronic diseases; CI, confidence interval; OR, odds ratio

**Fig. 3:**
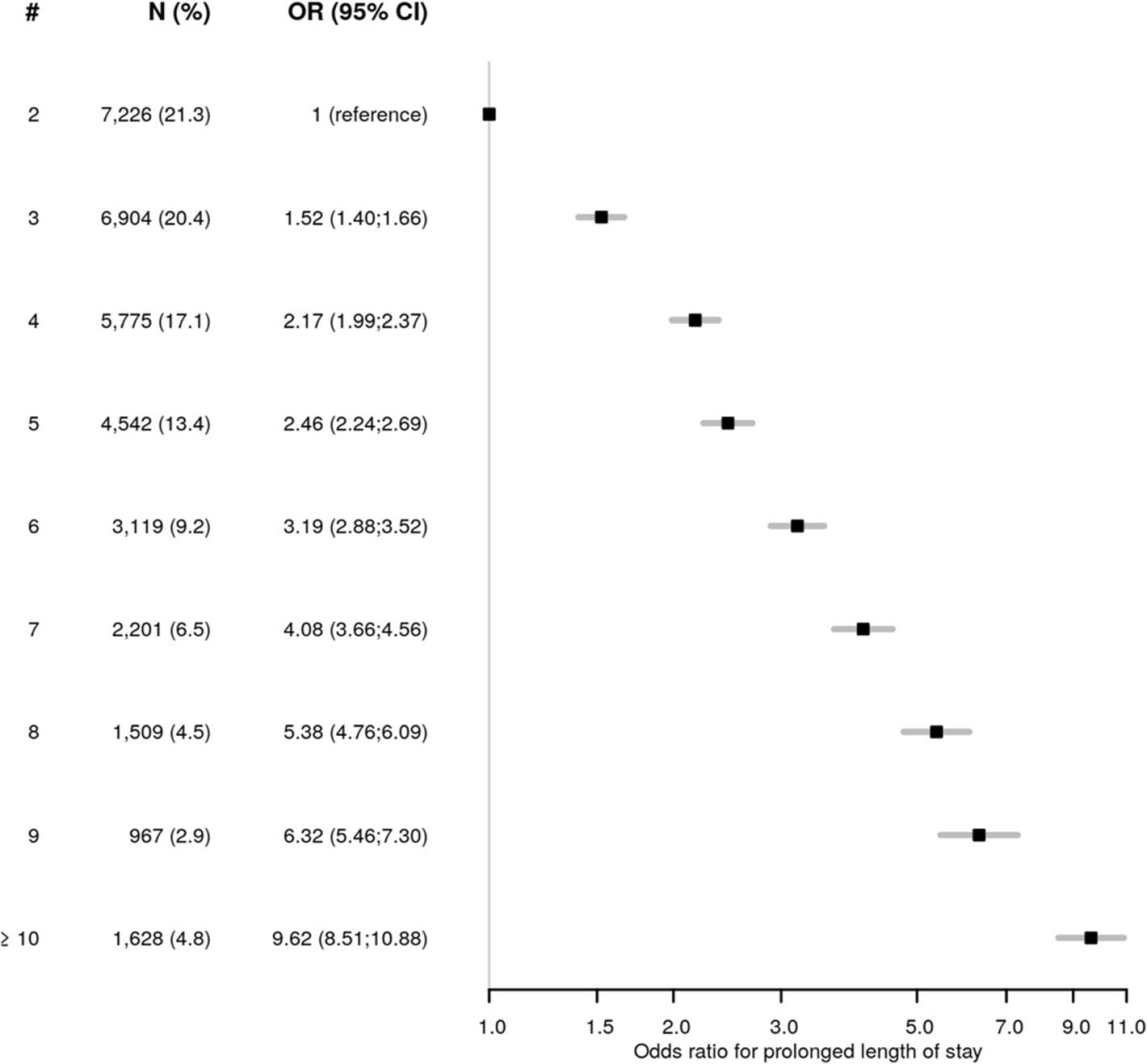
Association between the number of chronic diseases and prolonged length of stay. Legend: Odds ratio (box) with 95% confidence interval (lines) for prolonged length of stay. The reference category is the presence of 2 chronic diseases, as we included only patients with multimorbidity. A prolonged length of stay was defined as a stay longer than or equal to the upper quartile (75%). Abbreviations: #, number of chronic diseases; CI, confidence interval; OR, odds ratio

The odds for PAR increased with each additional chronic disease, reaching an OR of 2.31 (95%CI 1.87;2.85) for patients with 10 or more chronic diseases (Fig. 2). The effect on prolonged LOS was more pronounced, reaching an OR of 9.62 (95% CI 8.51;10.88) in patients with 10 or more chronic diseases (Fig. 3).

## Discussion

In this large sample of medical patients hospitalised in Switzerland, we identified combinations of chronic comorbidities associated with higher healthcare resource utilization, measured as PAR and prolonged LOS. Among the 20 combinations of comorbidities with the highest odds for PAR, 10 included CKD. These combinations increased the odds for PAR by 92 to 169%. Miscellaneous mental health disorders combined with mood disorders, and diseases of white blood cells combined with hematological malignancy, were strongly associated with a prolonged LOS. The comorbidities of 5 of the 20 combinations with the highest odds for a prolonged LOS had a more than multiplicative effect on the odds. The number of chronic diseases was strongly associated with LOS. This quantitative description of combinations of chronic comorbidities with the highest impact on healthcare resource utilization may help to identify patients most likely to benefit from preventive interventions in Switzerland.

### Combinations of comorbidities and 30-day potentially avoidable readmission

CKD was often found among the combinations of comorbidities with the highest odds for PAR. Previous studies described an increased risk for readmission among patients with CKD, but little was known on which patients with CKD may be more vulnerable [26–29]. Our study identified 10 different chronic comorbidities that increased the odds for PAR when combined with CKD, with the highest odds for the combinations including liver disease, solid malignancy and chronic obstructive pulmonary disease and bronchiectasis. Paying particular attention to those comorbidities in patients with CKD, particularly when developing preventive interventions, may help to reduce the rate of PAR in this population.

Solid malignancy was also frequently found among these top combinations. As we included only PAR, we did not consider patients readmitted for planned chemotherapy or radiotherapy in the outcome. Therefore, our findings suggest that these readmissions were rather related to complications of treatment or frailty, which is frequent among those patients and associated with adverse health outcome such as readmission [30–32]. This report is important, as both frailty and complications of treatment could be targeted by preventive interventions.

### Combinations of comorbidities and length of stay

Mood disorders were often found among one fourth of the combinations of comorbidities with the highest odds for prolonged LOS, and had the highest impact on the LOS in combination with miscellaneous mental health disorders. Previous studies associated psychological or psychiatric comorbidity with prolonged LOS in general hospital settings [33, 34]. In our study, we further found that a combination of psychological or psychiatric comorbidities was particularly strongly associated with prolonged LOS. Additionally, the two comorbidities had a multiplicative effect on the odds for a prolonged LOS. As miscellaneous mental health disorders include mostly sleep disorders not related to a somatic condition, our finding underlines the importance of paying attention to minimize factors associated with sleep disturbance during hospital stay, and particularly in patients with a preexisting psychiatric comorbidity, as they seem more vulnerable.

The second combination with the highest impact on LOS was diseases of white blood cells with hematological malignancy. This could be mainly explained by the neutropenia either resulting from chemotherapy or directly related to hematological malignancy [35]. Unfortunately, we may have only little impact on the prolonged LOS in this case, as neutropenic patients most often have to stay in an isolated hospital room, and because of the frequent and hardly avoidable complications of neutropenia, including severe mucositis and infections.

Neurological disorders were also frequent among the top combinations associated with a prolonged LOS. This finding is important, as different interventions, depending on the type of neurological disorder, may impact the LOS. On the one hand, in patients with cerebrovascular diseases, care by neurohospitalists or in stroke units have been shown to shorten the LOS [36, 37]. On the other hand, disability resulting from neurological disorders, such as paralysis, increases the need for supportive care after discharge, which may require some time to organize because of limited available resources. This is important, considered that a modification in resource allocation may decrease this adverse impact on the LOS.

Until now, we lacked data on how the different comorbidities may interact to influence the LOS. In this study, we found that the comorbidities of one fourth of the combinations with the highest OR for a prolonged LOS showed a more than multiplicative effect on the odds. This suggests that assessing multimorbidity as a count or a list of diseases only may mask important additional information on the higher healthcare resource utilization associated with specific combinations of comorbidities, and thus not exactly reflect the complex impact of multimorbidity on the LOS in particular.

### Number of chronic diseases and healthcare resource utilization

A prolonged LOS and a higher rate of readmission had been associated with the number of diseases, but we lacked quantitative data on this association [4, 15, 17, 18, 38]. Furthermore, little is known on this association with PAR, rather than with any readmission. Interestingly, the number of chronic diseases was far more strongly associated with LOS than with PAR, reaching an OR of 9.62 for prolonged LOS, but of only 2.31 for PAR in patients with 10 or more chronic diseases. This suggests that the number of chronic diseases may increase patients’ level of complexity, which has in turn been associated with prolonged LOS [39, 40]. The number of chronic diseases may thus help to identify patients that may benefit more of interventions aimed at shortening LOS. On the other hand, the association between the number of chronic diseases and PAR was less strong. This suggests that particular types of diseases, rather than the number of diseases, are associated with an increased odds for PAR. This hypothesis is supported by previous data finding a stronger association between PAR and some diseases, such as infection, neoplasm, heart failure or gastrointestinal and liver disorders [41]. The number of chronic diseases alone may therefore be less useful to allocate interventions aimed at reducing PAR.

### Limitations and strengths

Our study presents some limitations. First, although using ICD codes allowed assessing a broad number of diseases, diagnoses are subject to coding quality that may lead to underreporting. Second, we assessed diagnoses using hospital records only, which may have led to some biases in morbidity measurement. However, diagnoses are not coded with the ICD system in primary care settings in Switzerland, thus not allowing their classification with the Chronic Condition Indicator and the Clinical Classification Software. Third, as our study was not designed to assess causal relationships, we can only report associations. Fourth, our findings may have limited generalizability to patients hospitalised in primary or secondary care hospitals that may be less severely ill than those cared for in tertiary university teaching hospitals.

This study has several strengths also. First, we used a large and multicentre sample of patients. Second, we classified the diseases using standardised tools and considered all ICD-coded diagnoses, unlike most previous studies that often included a limited number of diseases [4, 22, 24, 42–45]. Finally, we used innovative methods to measure multimorbidity and its impacts on healthcare resource utilization, such as the assessment of combinations of comorbidities and of interactions between the comorbidities.

## Conclusions

In this large multicentre analysis of multimorbid medical inpatients, we found that half of the combinations of comorbidities most strongly associated with PAR included CKD and one third of them included solid malignancy. This should prompt physicians and researchers to develop specific preventive interventions particularly focused on these groups of patients, with the aim to reduce the risk of PAR and thus the burden of disease for patients with CKD or solid malignancy. Miscellaneous mental health disorders combined with mood disorders, and diseases of white blood cells combined with hematological malignancy, were the most strongly associated with LOS, with a difference in median LOS of 17.4 and 15.7 days, respectively. Hospital physicians caring for medical patients should thus be particularly attentive to identify and take care of mental health conditions, particularly sleep disorders, as these comorbidities may easily go unnoticed, but are associated with higher healthcare utilization. The number of chronic diseases was far more strongly associated with prolonged LOS than with PAR. This measure of multimorbidity may thus be more useful to identify patients at risk of prolonged LOS that may most benefit of interventions to shorten LOS.

In conclusion, this detailed and quantitative description may help healthcare providers to identify patients with combinations of diseases associated with higher healthcare resource utilization, and thus to better focus preventive interventions in order to reduce PAR and shorten LOS, with the overall objective to decrease healthcare costs and adverse consequences of multimorbidity for the patients, without decreasing quality of care.

## Supplementary information

**Additional file 1.** Multimorbidity and healthcare resource utilization in Switzerland: a multicentre cohort study.

## Data Availability

All data generated or analysed during this study are included in this published article and its supplementary information files.

## Abbreviations

CI: Confidence interval
CKD: Chronic kidney disease
ICD: International classification of diseases
IQR: Interquartile range
LOS: Length of stay
OR: Odds ratio
PAR: Potentially avoidable readmission
