# Supplementary material for: Multimorbidity and healthcare resource utilization in Switzerland: a multicentre cohort study
Source: BMC Health Serv Res. 2019 Oct 17;19:708. doi: 10.1186/s12913-019-4575-2 (PMC6798375; doi:10.1186/s12913-019-4575-2)
Supplement: Supplementary file 1 — Additional file 1. Multimorbidity and healthcare resource utilization in Switzerland: a multicentre cohort study. [file 12913_2019_4575_MOESM1_ESM.docx]

**Multimorbidity and healthcare resource utilization in Switzerland:**

a multicentre cohort study

**Appendix**

**Categorization of diseases**

For clinical relevance, we further merged together some categories of the Clinical Classification Software (CCS), creating following broader categories:

1. chronic heart disease (CHD): CCS 105-107 (cardiac dysrhythmias), CCS 100-101 (coronary heart disease), CCS 10 (nonhypertensive congestive heart failure), CCS 96 (heart valve disorder);
2. cerebrovascular diseases: CCS 109 and 111-112;
3. solid malignancies: CCS 11-36 and 41-42;
4. hematological malignancies: CCS 37-40;
5. arthropathy and arthritis: CCS 54 (gout and other crystal arthropathies), CCS 201 (infective arthritis and osteomyelitis), CCS 202 (rheumatoid arthritis and related disease), CCS 203 (osteoarthritis), CCS 204 (other non-traumatic joint disorders), CCS 205 (spondylosis, intervertebral disc disorders and other back problems);
6. osteoporosis and pathological fractures: CCS 206-207;
7. liver disease: CCS 6 and 150-151;
8. psychosis and schizophrenic disorders: CCS 70-71 and 659;
9. other nutritional, endocrine or metabolic disorder: CCS 51 and 58;
10. substance-related disorders: CCS 660-661.

**Appendix Table 1.** Clinical Classification Software (CCS) categories and their frequency in the total population. Data are number with percentage. Not all 285 CCS categories were present in the sample.

| **CCS Category** | **Total population (n=33,871)** |
| --- | --- |
| Chronic heart disease | 15,717 (46.40) |
| Solid malignancy | 4,964 (14.66) |
| Chronic kidney disease | 5,174 (15.28) |
| Substance-related disorders | 4,153 (12.26) |
| COPD and bronchiectasis | 2,979 (8.80) |
| Cerebrovascular disease | 2,843 (8.39) |
| Other nervous system disorders | 2,743 (8.10) |
| Arthropathy and arthritis | 2,479 (7.32) |
| Mood disorders | 2,470 (7.29) |
| Pulmonary heart disease | 2,161 (6.38) |
| Hematological malignancy | 1,921 (5.67) |
| Acute and unspecified renal failure | 2,015 (5.95) |
| Paralysis | 2,052 (6.06) |
| Peripheral and visceral atherosclerosis | 2,020 (5.96) |
| Thyroid disorders | 1,892 (5.59) |
| Dementia | 1,751 (5.17) |
| Liver disease | 1,570 (4.64) |
| Nephritis, nephrosis and renal sclerosis | 1,473 (4.35) |
| Epilepsy; convulsions | 1,337 (3.95) |
| Diseases of white blood cells | 1,381 (4.08) |
| Anxiety disorders | 1,315 (3.88) |
| Esophageal disorders | 1,272 (3.76) |
| Osteoporosis | 1,203 (3.55) |
| Other hereditary and degenerative nervous system conditions | 1,039 (3.07) |
| Other and ill-defined heart disease | 1,074 (3.17) |
| Miscellaneous mental health disorders | 932 (2.75) |
| Other nutritional/endocrine/metabolic disorders | 838 (2.47) |
| Systemic lupus erythematosus and connective tissue disorders | 720 (2.13) |
| Cardiac and circulatory congenital anomalies | 664 (1.96) |
| Chronic ulcer of skin | 701 (2.07) |
| Hyperplasia of prostate | 666 (1.97) |
| Asthma | 573 (1.69) |
| Parkinson`s disease | 536 (1.58) |
| Aortic; peripheral; and visceral artery aneurysms | 588 (1.74) |
| Occlusion or stenosis of precerebral arteries | 597 (1.76) |
| Other circulatory disease | 511 (1.51) |
| Retinal detachments; defects; vascular occlusion; and retinopathy | 488 (1.44) |
| Immunity disorders | 484 (1.43) |
| Diverticulosis and diverticulitis | 404 (1.19) |
| Other lower respiratory disease | 380 (1.12) |
| Biliary tract disease | 350 (1.03) |
| Personality disorders | 364 (1.07) |
| Glaucoma | 253 (0.75) |
| Other inflammatory condition of skin | 280 (0.83) |
| Multiple sclerosis | 196 (0.58) |
| Other diseases of kidney and ureters | 297 (0.88) |
| Aortic and peripheral arterial embolism or thrombosis | 268 (0.79) |
| Psychosis and schizophrenic disorders | 269 (0.79) |
| Regional enteritis and ulcerative colitis | 207 (0.61) |
| Cataract | 152 (0.45) |
| Neoplasms of unspecified nature or uncertain behavior | 242 (0.71) |
| Other diseases of veins and lymphatics | 229 (0.68) |
| Gastritis and duodenitis | 227 (0.67) |
| Other ear and sense organ disorders | 228 (0.67) |
| HIV infection | 212 (0.63) |
| Gastroduodenal ulcer (except hemorrhage) | 225 (0.66) |
| Adjustment disorders | 213 (0.63) |
| Headache; including migraine | 184 (0.54) |
| Other eye disorders | 183 (0.54) |
| Other diseases of bladder and urethra | 208 (0.61) |
| Other connective tissue disease | 171 (0.50) |
| Other congenital anomalies | 162 (0.48) |
| Other upper respiratory infections | 138 (0.41) |
| Other upper respiratory disease | 132 (0.39) |
| Other bone disease and musculoskeletal deformities | 118 (0.35) |
| Other hematologic conditions | 120 (0.35) |
| Malignant neoplasm without specification of site | 117 (0.35) |
| Cystic fibrosis | 104 (0.31) |
| Other acquired deformities | 106 (0.31) |
| Other gastrointestinal disorders | 99 (0.29) |
| Pancreatic disorders (not diabetes) | 96 (0.28) |
| Developmental disorders | 94 (0.28) |
| Inflammation; infection of eye (except that caused by tuberculosis or sexually transmitted disease) | 50 (0.15) |
| Genitourinary congenital anomalies | 81 (0.24) |
| Sickle cell anemia | 45 (0.13) |
| Other and unspecified benign neoplasm | 73 (0.22) |
| Allergic reactions | 59 (0.17) |
| Other CNS infection and poliomyelitis | 60 (0.18) |
| Nervous system congenital anomalies | 59 (0.17) |
| Menstrual disorders | 41 (0.12) |
| Inflammatory conditions of male genital organs | 36 (0.11) |
| Lung disease due to external agents | 35 (0.10) |
| Phlebitis; thrombophlebitis and thromboembolism | 32 (0.09) |
| Viral infection | 31 (0.09) |
| Tuberculosis | 30 (0.09) |
| Digestive congenital anomalies | 28 (0.08) |
| Other male genital disorders | 25 (0.07) |
| Disorders of teeth and jaw | 24 (0.07) |
| Prolapse of female genital organs | 25 (0.07) |
| Joint disorders and dislocations; trauma-related | 23 (0.07) |
| Attention-deficit, conduct, and disruptive behavior disorders | 21 (0.06) |
| Urinary tract infections | 21 (0.06) |
| Other female genital disorders | 21 (0.06) |
| Acquired foot deformities | 20 (0.06) |
| Other infections; including parasitic | 17 (0.05) |
| Disorders usually diagnosed in infancy, childhood, or adolescence | 15 (0.04) |
| Other disorders of stomach and duodenum | 16 (0.05) |
| Menopausal disorders | 16 (0.05) |
| Conditions associated with dizziness or vertigo | 12 (0.04) |
| Noninfectious gastroenteritis | 14 (0.04) |
| Otitis media and related conditions | 12 (0.04) |
| Acute and chronic tonsillitis | 11 (0.03) |
| Other skin disorders | 11 (0.03) |
| Sexually transmitted infections (not HIV or hepatitis) | 10 (0.03) |
| Impulse control disorders, NEC | 6 (0.02) |
| Encephalitis (except that caused by tuberculosis or sexually transmitted disease) | 5 (0.01) |
| Spinal cord injury | 4 (0.01) |
|  |  |

**Abbreviations**: COPD, chronic obstructive pulmonary disease.
